# Association of common genetic variation in the protein C pathway genes with clinical outcomes in acute respiratory distress syndrome

**DOI:** 10.1186/s13054-016-1330-5

**Published:** 2016-05-23

**Authors:** Anil Sapru, Kathleen D. Liu, Joseph Wiemels, Helen Hansen, Ludmilla Pawlikowska, Annie Poon, Eric Jorgenson, John S. Witte, Carolyn S. Calfee, Lorraine B. Ware, Michael A. Matthay, the NHLBI ARDS Network

**Affiliations:** Departments of Pediatrics, University of California, Box 0106, , 550, 16th Street, San Francisco, CA 94143 USA; David Geffen School of Medicine, Department of Pediatrics, University of California, 10833 Le Conte Avenue, 12-488 MDCC, Los Angeles, 90095 CA USA; Department of Medicine, University of California, San Francisco, CA USA; Department of Anesthesia and Perioperative Care, University of California, San Francisco, CA USA; Institute for Human Genetics, University of California, San Francisco, CA USA; Department of Medicine, Vanderbilt University, Nashville, TN USA; Cardiovascular Research Institute, University of California, San Francisco, CA USA

## Abstract

**Background:**

Altered plasma levels of protein C, thrombomodulin, and the endothelial protein C receptor are associated with poor clinical outcomes in patients with acute respiratory distress syndrome (ARDS). We hypothesized that common variants in these genes would be associated with mortality as well as ventilator-free and organ failure-free days in patients with ARDS.

**Methods:**

We genotyped linkage disequilibrium-based tag single-nucleotide polymorphisms in the ProteinC, Thrombomodulin and Endothelial Protein C Reptor Genes among 320 self-identified white patients of European ancestry from the ARDS Network Fluid and Catheter Treatment Trial. We then tested their association with mortality as well as ventilator-free and organ-failure free days.

**Results:**

The GG genotype of rs1042580 (*p* = 0.02) and CC genotype of rs3716123 (*p* = 0.002), both in the thrombomodulin gene, and GC/CC genotypes of rs9574 (*p* = 0.04) in the endothelial protein C receptor gene were independently associated with increased mortality. An additive effect on mortality (*p* < 0.001), ventilator-free days (*p* = 0.01), and organ failure-free days was observed with combinations of these high-risk genotypes. This association was independent of age, severity of illness, presence or absence of sepsis, and treatment allocation.

**Conclusions:**

Genetic variants in thrombomodulin and endothelial protein C receptor genes are additively associated with mortality in ARDS. These findings suggest that genetic differences may be at least partially responsible for the observed associations between dysregulated coagulation and poor outcomes in ARDS.

**Electronic supplementary material:**

The online version of this article (doi:10.1186/s13054-016-1330-5) contains supplementary material, which is available to authorized users.

## Background

Acute respiratory distress syndrome (ARDS) is a common cause of respiratory failure characterized by acute pulmonary edema and lung inflammation [1]. ARDS occurs in both adults and children and has an incidence of approximately 200,000 patients per year in the United States. Estimates of mortality range from 18 % to 58 % [2, 3]. The majority of deaths among patients with ARDS are attributed to multiorgan failure [1, 4].

A number of experimental and human studies suggest that excessive activation of coagulation is associated with increased mortality in patients with ARDS [5–11]. Activated protein C (PC) is an endogenous regulator of coagulation that has both anticoagulant and anti-inflammatory effects [12, 13]. Protein C is activated by thrombin in the presence of thrombomodulin (TM), and membrane-bound endothelial protein C receptor (EPCR) potentiates this activation [14]. Human lung epithelial cells express protein C, EPCR, and TM, and the lung epithelium can actively modulate the protein C pathway [15]. Alterations in plasma levels of protein C, TM, and soluble EPCR are associated with increased mortality and greater severity of illness among patients with ARDS [13, 16–18]. It is unclear if these alterations and their associations with clinical outcomes are determined exclusively by environmental factors that precipitate ARDS, such as the virulence of the infection, the extent of aspiration, and the severity of shock, or whether they are also influenced by genetic variation.

Common genetic variation (e.g., polymorphisms with minor allele frequency >5 %) in the genes encoding for PC, EPCR, and TM has been well-characterized and is associated with adverse clinical outcomes in disorders such as sepsis and cardiovascular disease [19–29]. Since the genetics of diseases such as ARDS are likely complex and therefore likely to involve several low-penetrance loci [30, 31], analyzing multiple variants of genes encoding components of the same physiological cascade may prove to be a more powerful approach than studies of single candidates [32–35]. We hypothesized that common genetic variations in the genes encoding for protein C, EPCR, and TM are associated with adverse clinical outcomes in patients with ARDS. We also hypothesized that variants in each individual gene may each have a small effect and that a combination of these variants would be associated with adverse clinical outcomes.

## Methods

### Study population

The study population included subjects enrolled in the ARDS Network Fluid and Catheter Treatment Trial (FACTT) from whom DNA was available. FACTT was a multicenter trial in which researchers compared conservative and liberal strategies of fluid management using explicit protocols applied for 7 days in patients with ARDS [36, 37]. Participants were also randomly assigned to receive either a pulmonary arterial catheter or a central venous catheter in a two-by-two factorial design [36]. All patients were ventilated using a lung-protective ventilation strategy. The primary outcome was mortality at 60 days [36, 37].

As part of the primary enrollment in the FACTT trial, patients were also asked to co-enroll in an ancillary study designed to study the role of genetic biomarkers. Patients who consented to participate in the ancillary study had additional whole blood collected, from which DNA was extracted. DNA was extracted and made available for this study by the ARDS Network DNA repository in the Center for Human Genetics Research at Vanderbilt University (Nashville, TN, USA). DNA was available from 470 patients, 320 of whom were of self-identified white race of European ancestry. We limited the present analysis to white patients of European ancestry to avoid confounding due to population stratification.

The institutional review boards of each participating hospital reviewed and approved the primary and ancillary studies. Written informed consent was obtained from participants or their legally authorized surrogates.

### Outcome measures

The primary outcome measure was mortality at 60 days. The secondary outcome measures were (1) the number of ventilator-free days (VFDs) [38] and (2) the number of organ failure-free days during the first 28 days of hospitalization [36].

### Single-nucleotide polymorphism selection and genotyping

To comprehensively characterize all the common genetic variation in these genes, we genotyped linkage disequilibrium (LD)-based single-nucleotide polymorphisms (tag SNPs) in the genomic region and 2000 bp upstream and downstream in the PC, EPCR, and TM genes. We used the resequencing data on these genes available from the Seattle SNPs website (http://snp.gs.washington.edu/SeattleSeqAnnotation144/) and selected tag SNPs using MULTIPOP software [39] with minimum allele frequency set at 5 % and *r*^2^ set at 0.8.

SNPs were genotyped using three commercially available technologies according to the manufacturers’ instructions: Illumina Golden Gate 384-plex (Illumina, San Diego, CA, USA), GenomeLab SNPstream 48-plex (Beckman Coulter, Brea, CA, USA), and template-directed primer extension with fluorescence polarization detection using the AcycloPrime II kit (PerkinElmer, Waltham, MA, USA) [40]. Primer sequences are available upon request. Samples were arrayed on 96-well plates with negative and positive controls (duplicates) on each plate. Investigators blinded to the clinical status scored the genotypes.

### Data analysis

Nonpolymorphic SNPs and SNPs with minor allele frequency <5 % were removed from the analysis. All analyzed SNPs were tested and found to be in Hardy-Weinberg equilibrium. We assessed genotypic effects at single SNP loci in each of the three genes using the χ^2^ test to compare the associations of genotypes with 60-day mortality. In the univariate analysis of individual SNP genotypes, additive, dominant, and recessive models were considered. Next, we used multivariate logistic regression, incorporating the associated SNPs in a single multivariate model to assess if each SNP was independently associated with mortality when tested with other SNPs in the same gene. We included age, presence of nonpulmonary sepsis, fluid management strategy, and Acute Physiology and Chronic Health Evaluation (APACHE) score as covariates in the model because of their previously reported association with clinical outcomes in patients with ARDS. We corrected for multiple comparisons for the multiple SNPs genotyped within each gene by using multiple permutations as implemented in PLINK [41].

SNPs from each gene with an independent effect on mortality were retested in a logistic regression model with the covariates mentioned above and all SNPs with a statistically significant association with outcome. We also conducted an exploratory analysis, testing for a haplotype effect. Haplotype frequencies in each gene were estimated from unphased genotype data using the PHASE algorithm in Haploview, and the association of the imputed haplotypes on mortality was assessed using a case-control approach as implemented in Haploview [42, 43].

We examined the joint effect of a combination of these “high-risk genotypes” associated with mortality on clinical outcomes. For the purpose of this analysis, we defined the following genotypes with an independent effect on mortality as high-risk genotypes:

1. CC genotype of the rs3176123 SNP in the TM gene
2. GG genotype of the rs1042580 SNP in the TM gene
3. GC/GG genotypes of the rs9574 SNP in the EPCR gene

The association of combinations of genotypes with outcomes was assessed using the Cochran-Armitage trend test to compare clinical outcomes across categories of patients stratified by the number of high-risk genotypes possessed by each individual. We used regression models to adjust for age, severity of illness (APACHE), presence of sepsis, and allocation to treatment arm. In addition, given the a priori hypothesis that all three genes in the protein C pathway (i.e., protein C, EPCR, and TM) would have an effect on mortality in patients with ARDS, we conducted an additional analysis with the rs1799810 SNP of the protein C gene included in the combined model.

All analyses were carried out using Stata 9 (StataCorp, College Station, TX, USA), PLINK [42], and Haploview [43, 44] software. On the basis of an assumption of minimum allele frequency of 5 % and a dominant model, the sample size of 320 patients has a power of 80 % to detect an increase in mortality by a relative ratio of 2.1 or greater at *p* < 0.05.

## Results

The baseline characteristics of the self-identified white patients of European ancestry enrolled in the FACTT trial stratified by the 320 for whom DNA was available and the 321 for whom DNA was not available are depicted in Table 1. The baseline characteristics of the study population are similar to the white patients of European ancestry in the FACTT trial for whom DNA was not available. Genotype frequencies of the assayed SNPs in the protein C, EPCR, and TM genes and the frequencies stratified by mortality at 60 days are depicted in Tables 2, 3 and 4.

**Table 1:** Characteristics of patients from the Fluid and Catheter Treatment Trial

Variable	Caucasians with DNA (*n* = 320)	Caucasians without DNA (*n* = 321)	*p* Value
Age, years, mean ± SD	50 ± 16	52 ± 16	0.06
Male sex, %	51 %	51 %	0.96
APACHE III score, mean ± SD	92 ± 29	92 ± 31	0.77
Clinical disorders associated with acute lung injury			
Pneumonia, %	46 %	47 %	0.78
Trauma, %	8 %	5 %	0.15
Sepsis, %	21 %	25 %	0.26
Multiple transfusions, %	2 %	1 %	0.25
Aspiration, %	17 %	17 %	0.90
Baseline PaO_2_/FiO_2_	103 ± 48	106 ± 49	0.45
Severity of ARDS			0.36
Mild	59 %	53 %	
Moderate	36 %	41 %	
Severe	5 %	6 %	
Day 1 PIP	32 ± 8	32 ± 9	0.32
Day 1 PEEP	9.4 ± 4	9.5 ± 4	0.7
Fluid management, liberal, %	48 %	55 %	0.09
Mortality at 60 days, %	21 %	25 %	0.19
Ventilator-free days, median (range)	19 (0–28)	17 (0–28)	0.22

*APACHE* Acute Physiology and Chronic Health Evaluation, *ARDS* acute respiratory distress syndrome, *FiO*_*2*_ fraction of inspired oxygen, *PaO*_*2*_ partial pressure of arterial oxygen, *PEEP* positive end-expiratory pressure, *PIP* peak inspiratory pressure

**Table 2:** Genotype counts of protein C tag single-nucleotide polymorphisms among survivors and nonsurvivors

dbSNP ID	Allele 1	Allele 2	Genotype counts^a^ (nonsurvivors)	Genotype counts^a^ (survivors)	*p* Value
rs1158867	C	T	8/23/19	32/91/55	0.34
rs1799808	T	C	9/31/25	27/105/117	0.21
rs1799810	T	A	10/31/26	47/129/76	0.17
rs2069901	G	A	10/31/26	46/128/78	0.22
rs2069904	A	G	7/26/34	31/108/114	0.40
rs2069910	A	G	16/29/22	51/112/87	0.53
rs2069912	G	A	6/25/36	23/99/129	0.73
rs2069914	A	G	6/25/36	22/100/129	0.73
rs2069916	A	G	9/31/27	26/104/118	0.26
rs2069918	A	G	5/23/39	17/86/149	0.83
rs2069920	G	A	14/29/24	36/116/97	0.19
rs2069924	A	G	9/32/26	27/106/116	0.25
rs2069928	A	C	3/21/43	10/72/168	0.64
rs2069931	A	G	10/29/28	31/106/114	0.50
rs2069933	A	G	8/29/30	35/102/115	0.67
rs5937	G	A	7/26/34	26/109/117	0.52
rs908787	G	C	1/4/62	3/25/224	0.46
rs971207	A	G	7/30/30	35/104/112	0.45

*dbSNP ID* single-nucleotide polymorphism database identifier

^a^Genotype counts (minor homozygotes/heterozygotes/major homozygotes)

**Table 3:** Genotype counts of endothelial protein C receptor tag single-nucleotide polymorphisms among survivors and nonsurvivors

dbSNP ID	Allele 1	Allele 2	Genotype counts^a^ (survivors)	Genotype counts^a^ (nonsurvivors)	*p* Value (uncorrected)	*p* Value^b^ (corrected)
rs2069948	G	A	8/40/19	58/116/75	0.05	0.07
rs2069951	A	G	0/7/60	0/24/229	0.76	1
rs2069952	G	A	7/40/19	58/116/77	0.03	0.05
rs867186	G	A	0/8/59	0/47/205	0.18	0.4
rs9574	C	G	7/38/19	58/111/75	0.02	0.04

*dbSNP ID* single-nucleotide polymorphism database identifier

^a^Genotype counts (minor homozygotes/heterozygotes/major homozygotes)

^b^ *p* Value (corrected) is corrected for multiple SNPs tested within the endothelial protein C receptor gene

**Table 4:** Genotype counts of thrombomodulin tag single-nucleotide polymorphisms among survivors and nonsurvivors

dbSNP ID	Allele 1	Allele 2	Genotype counts^a^ (nonsurvivors)	Genotype counts^a^ (survivors)	*p* Value (uncorrected)	*p* Value^b^ (corrected)
rs1042580	G	A	17/29/21	32/127/93	0.010	0.02
rs1962	G	A	2/20/45	19/85/149	0.18	0.39
rs2007363	A	C	1/18/48	15/77/161	0.14	0.26
rs3176118	O	X	1/9/55	2/45/204	0.58	1
rs3176123	C	A	8/11/48	6/76/169	0.0001	0.002

*dbSNP ID* single-nucleotide polymorphism database identifier

^a^Genotype counts (minor homozygotes/heterozygotes/major homozygotes)

^b^ *p* Value (corrected) is corrected for multiple SNPs tested within thrombomodulin gene

In the EPCR gene, the GC/GG genotypes of the rs9574 SNP were associated with higher mortality (24 % vs. 11 %) compared with the CC genotype (*p* = 0.04 after correction for multiple SNPs tested in the EPCR gene). Two other SNPs in the EPCR gene—rs2069952 and rs2069948—that were highly correlated with rs9574 (*r*^2^ = 0.95), were also associated with mortality at 60 days; however, when tested in a regression model that included these two SNPs and rs9574, only rs9574 retained the association with mortality, suggesting that the effect was not independent of the effect of the rs9574 SNP. The association of the rs9574 SNP with mortality was independent of age, severity of illness, sepsis as the primary cause of ARDS, and the fluid management arm (Table 5).

**Table 5:** Multivariate logistic regression model with mortality at 60 days as the outcome and genotypes as predictors

Predictors	OR	95 % CI	*p* Value
Endothelial protein C receptor (EPCR) SNPs			
rs9574, GC/GG vs. CC	2.8	1.1–7.3	0.04
Age, years	1.03	1.01–1.05	<0.003
APACHE III score	1.04	1.02–1.05	<0.001
Sepsis, yes/no	1.3	0.6–2.7	0.6
PaO_2_/FiO_2_	1.001	0.99–1.01	0.7
Fluid strategy, liberal vs. conservative	1.5	0.8–2.8	0.24
Thrombomodulin (TM) SNPs			
rs3176123, CC vs. AA/AC	6.3	1.6–24.8	0.008
rs1042580, GG vs. AA/AG	2.8	1.2–6.1	<0.02
Age, years	1.02	1.00–1.05	0.03
APACHE III score	1.04	1.02–1.05	<0.001
Sepsis, yes/no	1.2	0.6–2.3	0.55
PaO_2_/FiO_2_	1.001	0.99–1.01	0.15
Fluid strategy, liberal vs. conservative	1.2	0.6–2.3	0.88
Thrombomodulin, endothelial protein C receptor and protein C SNPs (combined model)			
THBD rs3176123	6.1	1.6–24	0.01
THBD rs1042580	2.6	1.1–6.1	0.03
EPCR rs9574	2.9	1.08–7.9	0.03
Age, years	1.03	1.0–1.05	<0.02
APACHE III score	1.04	1.01–1.05	<0.001
Sepsis, yes/no	1.3	0.6–2.7	0.53
PaO_2_/FiO_2_	1.00	0.99–1.01	0.71
Fluid strategy, liberal vs. conservative	1.3	0.6–2.6	0.44

*APACHE* Acute Physiology and Chronic Health Evaluation, *EPCR* endothelial protein C receptor, *FiO*_*2*_ fraction of inspired oxygen, *PaO*_*2*_ partial pressure of arterial oxygen, *SNP* single-nucleotide polymorphism, *THBD* thrombomodulin gene, *TM* thrombomodulin

Models are adjusted for age, APACHE III score, presence of sepsis, baseline PaO_2_/FiO_2_ ratio, and allocation to fluid management arm

Two SNPs in the TM gene were independently associated with mortality at 60 days. The CC genotype of the rs3176123 SNP was associated with higher mortality (57 % vs. 20 %) compared with the AC/CC genotypes (*p* = 0.002 corrected for multiple SNPs tested). The GG genotype of the rs1042580 SNP was also associated with higher mortality (35 % vs. 19 %) compared with the AG/AA genotypes (*p* = 0.02 corrected for multiple SNPs tested). These two SNPs had limited correlation with each other (*r*^2^ = 0.14), and both SNPs were independently associated with mortality when analyzed in a joint logistic regression model including the two SNPS and age, presence of non-pulmonary sepsis, APACHE score, and allocation to fluid management strategy (Table 5). The association of the rs3176123 and rs1042580 SNPs with mortality was independent of age, severity of illness, sepsis, and the fluid management arm (Table 5). Of the protein C SNPs analyzed, the AA genotype of the rs1799810 SNP showed a trend toward increased mortality (26 % vs. 19 %) compared with the TT/AT genotypes, but this trend was not statistically significant (*p* = 0.18).

**Fig. 1:**
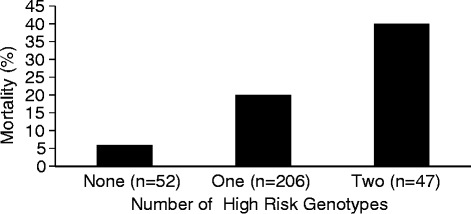
Patients are stratified on the basis of the number of high-risk genotypes possessed by each individual, and the height of the bars represents 60-day mortality in each group. There is a stepwise increase in mortality with increasing number of high-risk genotypes: none 5.8 % [1.2–16.2], *n* = 51; one 20 % [15–26], *n* = 200; and two 41 % [27–57], *n* = 46 (*p* < 0.001)

We tested the effect of a combination of these unfavorable SNPs on mortality at 60 days in a joint logistic regression model. On the basis of their biological interaction in a common pathway, we hypothesized that multiple unfavorable SNPs would have a more significant impact on the protein C pathway and therefore would be associated with increased mortality. The CC genotype of the rs3176123 SNP, the GG genotype of the rs1042580 SNP in the TM gene, and the GC/CC genotypes of rs9574 SNP in the EPCR gene were independently associated with mortality at 60 days. This association was independent of age, severity of illness, sepsis, and the fluid management arm (Table 5). We also examined mortality at 60 days among patients stratified by the number of high-risk genotypes possessed by each individual. There was an increase in mortality at 60 days with the presence of each additional high-risk genotype: 5.8 % [1.2–16.2], 20 % [15–26], and 41 % [27–57] (*p* < 0.001) (Fig. 1). The association was independent of age, severity of illness, sepsis, and the fluid management arm.

**Fig. 2:**
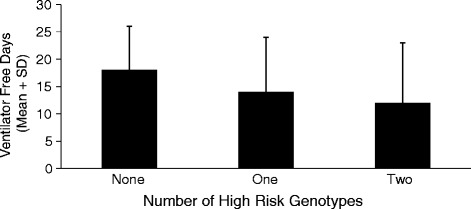
Patients are stratified on the basis of the number of high-risk genotypes possessed by each individual, and the height of the bars represents the number of ventilator-free days in each group. There is a stepwise decrease in the number of ventilator-free days with increasing number of high-risk genotypes (*p* = 0.02)

The number of VFDs was also compared among patients stratified by the number of high-risk genotypes carried by each individual. There was a decrease in the number of VFDs with the presence of each additional high-risk genotype (*p* = 0.01) (Fig. 2). This association was also independent of age, severity of illness, sepsis, and the fluid management arm.

**Fig. 3:**
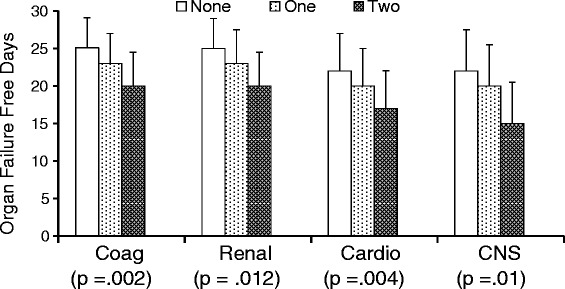
Patients are stratified on the basis of the number of high-risk genotypes possessed by each individual. Results are shown by organ system (i.e., coagulation [Coag], renal, cardiovascular [Cardio], and central nervous system [CNS]). The *y*-axis represents the number of organ failure-free days. There is a stepwise decrease in the number of organ failure-free days with increasing number of high-risk genotypes in all four organ systems (*p* values for each system are reported in parentheses along the *x*-axis)

The number of coagulation, renal, cardiovascular, and central nervous system organ failure-free days was compared among patients stratified by the number of high-risk genotypes carried by each individual. There was also a decrease in the number of coagulation, renal, cardiovascular, and central nervous system organ failure-free days with the presence of each additional high-risk genotype (Fig. 3).

Given our a priori hypothesis that genetic heterogeneity in all three genes (protein C, EPCR, and TM) in this pathway would have an effect on mortality in patients with ARDS, we conducted an additional analysis with four SNPs in the model: the three SNPs that are independently associated with mortality at 60 days and a fourth, the rs1799810 SNP in the protein C gene that had a trend toward increased mortality which did not achieve statistical significance. Even though the protein C SNP rs1799810 individually did not have a statistically significant association with mortality, we found that the mortality increased from 10.3 % [2.2–27.3] for those with no high-risk SNPs vs. 15.2 % [9.9–21.7], 28.5 % [19.8–38.5], and 63.6 % [30.8–89.1] for those with one, two, and three high-risk SNPs, respectively. Those with greater numbers of high-risk SNPs had fewer VFDs and fewer organ failure-free days (see Additional file 1). We did not find any joint haplotype effect on either the primary or secondary outcomes in any of these three genes.

### Thrombomodulin SNPs and plasma levels

Plasma levels of soluble TM were higher among individuals carrying the GG genotype of the rs1042580 SNP (median 106 ng/ml, interquartile range 75–187 ng/ml) as compared with the individuals with the AG and AA genotypes (median 90 ng/ml, interquartile range 59–141 ng/ml) (*p* < 0.05). The plasma TM levels among individuals with the CC genotype of the rs3176123 SNP (median 94 ng/ml, interquartile range 53–148 ng/ml) did not have a statistically significant difference from the individuals carrying the AC and CC genotypes (median 93 ng/ml, interquartile range 61–149 ng/ml) (*p* = 0.85).

### EPCR SNPs and plasma levels

Plasma levels of soluble EPCR were higher among individuals carrying the GG genotype of rs9574 SNP (median 66.1 ng/ml, Interquartile range 45–104 ng/ml) as compared with the individuals with the AG and AA genotypes (median 88.5 ng/ml, interquartile range 56–133 ng/ml) (*P* < 0.01).

To test whether the effect of the SNPs of the rs1042580 was mediated via plasma TM levels, we first tested the association of rs1042580 with mortality in a logistic regression model and then added plasma TM levels to the regression model. With addition of plasma TM to the regression model, the odds of mortality decreased slightly from 2.32 (95 % CI 1.06–5.1, *p* < 0.04) to 2.15 (95 % CI 0.93–4.9, *p* = 0.07), suggesting that plasma TM levels are at most a minor part of the effect of the SNP on mortality.

To test whether the effect of the SNPs of the rs9574 was mediated via plasma soluble Endothelial Protein C Receptor (sEPCR) levels, we tested the association of rs9574 with mortality in a logistic regression model and then added plasma EPCR as a mediator in the regression model. With addition of plasma sEPCR to the regression model, the odds of mortality decreased from 2.53 (95 % CI 1.1–5.8, *p* < 0.03) to 2.23 (95 % CI 0.96–5.2, *p* = 0.06), again suggesting that soluble Endothelial Protein C Receptor (EPCR) mediates a minor part of the effect of the rs9574 SNP on mortality.

## Discussion

The results of this study indicate that common genetic variations in the protein C pathway are associated with adverse clinical outcomes in adult patients with ARDS. The GC/CC genotypes of the rs9574 SNP in the EPCR gene, as well as the GG genotype of the rs1042580 SNP and the CC genotype of the rs3176123 SNP, both in the TM gene, were independently associated with mortality in ARDS. We also found that a combination of these high-risk genotypes had an additive effect on mortality, ventilator-free days, and organ failure-free days in ARDS. However, the effect of SNPs was not mediated via their effect on the levels of soluble TM or EPCR in plasma, suggesting the SNPs may have their effect through other mechanisms related to altered functioning of these molecules. This finding has major implications for current understanding of the pathogenesis of ARDS. Abnormalities of the coagulation pathway and its regulatory proteins and the association of these abnormalities with clinical outcomes in patients with ARDS have been described previously [6–11]. However, these abnormalities were thought to be due largely to environmental factors (e.g., severity of illness, virulence of organisms, severity of lung injury). The present findings suggest that genetic susceptibility may contribute to this dysregulated coagulation and the poor clinical outcomes in patients with ARDS.

Both intraalveolar and systemic coagulation are activated in patients with ARDS [8–10, 44]. Although intraalveolar fibrin deposition may have beneficial effects on gas exchange by sealing leakage sites and compartmentalizing infection, excessive fibrin deposition can be harmful because it can activate neutrophils and fibroblasts, compromise endothelial integrity, contribute to a loss of surfactant activity, decrease alveolar fluid clearance, and induce thrombotic obstruction of the microcirculation [6, 7]. The injury to the pulmonary microcirculation via inflammatory and thrombotic mechanisms may contribute to the increase in the pulmonary dead space fraction that is an independent predictor of mortality in ARDS [45]. In addition, systemic activation of coagulation may also contribute to hypercoagulability and the development of multiorgan failure with widespread microvascular thrombus formation [1, 4, 17].

Activated protein C is an endogenous regulator of coagulation that has both anticoagulant and anti-inflammatory effects. Alterations in plasma levels of protein C, EPCR, or TM that may contribute to decreased availability of activated protein C are associated with adverse clinical outcomes in patients with ARDS [16, 17]. In the present study, we examined the association of genetic variation in these protein C pathway genes with clinical outcomes in patients with ARDS. The results therefore add to the significance of the previously reported associations of alterations in these protein biomarker levels with protein C pathway proteins and adverse clinical outcomes, and they suggest that genetic predisposition may play a role in these previously reported associations of abnormalities in protein C pathway proteins and clinical outcomes in ARDS.

Although we chose a hypothesis-free approach within the candidate genes, the SNPs that were associated with adverse clinical outcomes in this study have all previously been reported (directly or indirectly via a tightly linked SNP) to have an association with protein levels and/or clinical outcomes in other conditions. The rs3176123 SNP in the TM gene is in tight LD with the rs1042579 SNP (*r*^2^ = 1), a coding region nonsynonymous SNP. The minor allele of this SNP is associated with increased incidence of venous thrombosis [46, 47]. In the present study, the minor allele homozygotes had increased mortality. The GG genotype of the rs1042580 SNP in the TM gene, which was associated with increased mortality in this study, has been associated with increased cardiovascular disease in females in combination with factor V Leiden in previous studies [32]. In our present study, the rs1042580 SNP was associated with variation in the soluble TM levels in plasma, but that explained only a minor part of the overall effect of the SNP on mortality, suggesting that the SNP may have its effect of through other mechanisms, including the possibility of altering the functional activity of TM. Finally, in a previous study, the haplotype tagged by the C allele of the rs9574 SNP in the EPCR gene was associated with increased levels of activated protein C levels and reduced risk of venous thromboembolism [21, 22]. In the present study, we found that the G allele at this locus is associated with increased mortality. The rs9574 SNP was associated with variation in the soluble EPCR levels in plasma, but that variation explained only a part of the overall effect of the SNP on mortality, suggesting that the SNP may have its effect through other mechanisms, including the possibility that the SNP may have an additional effect on the functional activity of EPCR.

A novel feature of this study is the combined analysis of the three genes. In a complex illness such as ARDS, the impact of genetic factors is likely to be determined by several variants of small effect size and their possible interactions. When acting together, these gene variants may affect the disease outcomes more profoundly than do the single predisposing variants [32]. An analysis comprising several genes that belong to the same pathway may reveal cumulative allelic effects (additive or multiplicative) as compared with single SNPs, which individually may have only a modest effect or no measurable impact on clinical outcomes. The combined analysis of these three proteins is based on the hypothesis that the three proteins of the protein C pathway act together to generate the final product (i.e., activated protein C, which might be the biologically active product responsible for the clinical effect). Given our a priori hypothesis that all three genes in this pathway would have an effect on mortality in patients with ARDS, we carried out an additional exploratory analysis using the rs1799810 SNP from the protein C gene based on the biological role of protein C in the common pathway, even though this SNP individually had only showed a trend toward association with increased mortality. Interestingly, the combination of high-risk genotypes from all three genes in the pathway was associated with mortality and adverse clinical outcomes upon addition of the protein C SNP (which was not independently associated with mortality) to the model.

There are several strengths of our study. These include the well-characterized clinical phenotype and the multicenter cohort of patients from a large, well-designed clinical trial. The previously reported association of clinical outcomes with protein levels and of the coagulation genes chosen for this study provides the biological plausibility for the candidate genes chosen for this study. All the SNPs associated with poor outcomes in ARDS in our study (or a tightly linked SNP) have been previously reported to affect protein levels and clinical outcomes in other populations [19–21, 26, 32, 48]. This finding further supports the biological plausibility and increases the prior probability for the reported associations. This point is important because genetic association studies with high prior probability are likely to have a low probability of reporting false-positive associations [49]. Finally, we found not only an association of genotypes with mortality but also a strong association with multiple-organ failure-free days. This association is significant because multiorgan failure is the most common attributable cause of mortality among patients with ARDS [1, 4]. This could be related to the fact that multiorgan failure and death are associated with each other; alternatively, the association of the high-risk genotypes with the number of organ failure-free days may suggest that multiorgan failure may be an intermediate phenotype, which in turn leads to the higher mortality associated with the high-risk genotypes.

This study also has some limitations. First, the results were obtained only in self-identified white patients of European ancestry. Second, we did not correct for study-wide multiple comparisons. Since our hypothesis was based on the biological plausibility of the three genes in the protein C pathway and previously reported associations with protein levels, we corrected for multiple comparisons for multiple SNPs tested within each gene rather than for study-wide multiple comparisons as is the case with hypothesis-free genome-wide association studies. Therefore, it is unlikely that these associations were due to chance alone. However, as is true for all genetic epidemiology studies, these findings need to be tested and validated in other patient cohorts.

## Conclusions

On the basis of this cohort, the GC/GG genotypes of the rs9574 SNP in the EPCR gene, as well as the CC genotype of rs3716123 and the GG genotype of the rs124580 SNPs in the TM gene, were associated with increased mortality in adults with ARDS. A combination of these genotypes had an additive effect on mortality, ventilator-free days, and organ failure-free days in patients with ARDS. These findings suggest that genetic differences may be at least partially responsible for the observed associations between dysregulated coagulation and poor outcomes in patients with ARDS. If confirmed, these findings may support the potential value of testing targeted therapies for ARDS in genetically predisposed patients.

## Additional file

Additional file 1: Figure S1. Patients are stratified on the basis of the number of high-risk genotypes (four-SNP model) each one possesses, and the height of the bars represents 60-day mortality in each group (none 10.3 % [2.2–27.3], *n* = 29; one 15.2 % [9.9–21.7], *n* = 158; two 28.5 % [19.8–38.5], *n* = 98; three 63.6 % [30.8–89.1], *n* = 11). There is a stepwise increase in mortality with increasing number of high-risk genotypes (*p* < 0.001). **Figure S2** Patients are stratified on the basis of the number of high-risk genotypes (four-SNP model) each one carries, and the height of the bars represents the number of ventilator-free days in each group. There is a stepwise decrease in the number of ventilator-free days with increasing number of high-risk genotypes (*p* = 0.01). **Figure S3** Patients are stratified on the basis of the number of high-risk genotypes (four-SNP model) each individual possesses. Results are shown by organ system (i.e., coagulation [Coag], renal, cardiovascular [Cardio], and central nervous system [CNS]). The *y*-axis represents the number of organ failure-free days. There is a stepwise decrease in the number of organ failure-free days with increasing number of high-risk genotypes in all four organ systems (*p* values for each system are reported in parentheses along the *x*-axis). **Table S1** Characteristics of protein C tag SNPs. **Table S2** Characteristics of EPCR tag SNPs. **Table S3** Characteristics of thrombomodulin gene tag SNPs. (DOC 1854 kb)

## Abbreviations

APACHE: Acute Physiology and Chronic Health Evaluation
ARDS: acute respiratory distress syndrome
CNS: central nervous system
EPCR: endothelial protein C receptor
FACTT: Fluid and Catheter Treatment Trial
FiO_2_: fraction of inspired oxygen
LD: linkage disequilibrium
PaO_2_: partial pressure of arterial oxygen
PC: protein C
PEEP: positive end-expiratory pressure
PIP: peak inspiratory pressure
SNP: single-nucleotide polymorphism
*THBD*: thrombomodulin gene
TM: thrombomodulin
VFD: ventilator-free day
