# Supplementary material for: Association of common genetic variation in the protein C pathway genes with clinical outcomes in acute respiratory distress syndrome
Source: Crit Care. 2016 May 23;20:151. doi: 10.1186/s13054-016-1330-5 (PMC4876559; doi:10.1186/s13054-016-1330-5)
Supplement: Additional file 1: Figure S1. — Patients are stratified on the basis of the number of high-risk genotypes (four-SNP model) each one possesses, and the height of the bars represents 60-day mortality in each group (none 10.3 % [2.2–27.3], n = 29; one 15.2 % [9.9–21.7], n = 158; two 28.5 % [19.8–38.5], n = 98; three 63.6 % [30.8–89.1], n = 11). There is a stepwise increase in mortality with increasing number of high-risk genotypes (p < 0.001). Figure S2 Patients are stratified on the basis of the number of high-risk genotypes (four-SNP model) each one carries, and the height of the bars represents the number of ventilator-free days in each group. There is a stepwise decrease in the number of ventilator-free days with increasing number of high-risk genotypes (p = 0.01). Figure S3 Patients are stratified on the basis of the number of high-risk genotypes (four-SNP model) each individual possesses. Results are shown by organ system (i.e., coagulation [Coag], renal, cardiovascular [Cardio], and central nervous system [CNS]). The y-axis represents the number of organ failure-free days. There is a stepwise decrease in the number of organ failure-free days with increasing number of high-risk genotypes in all four organ systems (p values for each system are reported in parentheses along the x-axis). Table S1 Characteristics of protein C tag SNPs. Table S2 Characteristics of EPCR tag SNPs. Table S3 Characteristics of thrombomodulin gene tag SNPs. (DOC 1854 kb) [file 13054_2016_1330_MOESM1_ESM.doc]

Legends for supplementary Figures

Supplementary Figure 1. Patients are stratified based on the number of High Risk Genotypes (Four SNP model) possessed by each individual and the height of the bars represents 60-day mortality in each group. None 10.3% [2.2-27.3] n = 29, One 15.2% [9.9-21.7] n= 158, Two 28.5% [19.8-38.5] n= 98, Three 63.6% [30.8-89.1] n=11. There is a stepwise increase in mortality with increasing number of High Risk Genotypes (p<0.001)

Supplementary Figure 2. Patients are stratified based on the number of High Risk Genotypes (Four SNP model) carried by each individual and the height of the bars represents the number of ventilator-free days in each group. There is a stepwise decrease in the number of ventilator-free days with increasing number of High Risk Genotypes (p = 0.01)

Supplementary Figure 3. Patients are stratified based on the number of High Risk Genotypes (Four SNP model) possessed by each individual. Results are shown by organ system, i.e, coagulation (Coag), renal, cardiovascular (Cardio) and central nervous system (CNS). The y axis represents the number of organ failure free days. There is a stepwise decrease in the number of organ-failure free days with increasing number of High Risk genotypes in all the four organ systems (p values for each system are reported in parentheses along the X- axis).

**Supplementary Figure 1**

**Supplementary Figure 2.**

**Supplementary Figure 3.**

Supplementary Table 1. Characteristics of Protein C tag- SNPs

| **Position on Chromosome 2** | **SNP** | **Allele 1** | **Allele 2** | **Genotype Counts* (Overall)** | **Minor Allele Frequency** | **Hardy-Wienberg (Chi-Square) P** |  | **Missingness (%)** |  |  |  |  |  |
| --- | --- | --- | --- | --- | --- | --- | --- | --- | --- | --- | --- | --- | --- |
| 127893848 | rs1158867 | C | T | 40/114/74 | 0.43 | 0.79 |  | 29 |  |  |  |  |  |
| 127892333 | rs1799808 | T | C | 36/136/142 | 0.33 | 0.70 |  | 1.5 |  |  |  |  |  |
| 127892510 | rs1799810 | T | A | 57/160/102 | 0.43 | 0.73 |  | 0 |  |  |  |  |  |
| 127891313 | rs2069901 | G | A | 56/159/104 | 0.42 | 0.81 |  | <0.04 |  |  |  |  |  |
| 127892249 | rs2069904 | A | G | 38/134/148 | 0.33 | 0.37 |  | 0 |  |  |  |  |  |
| 127894444 | rs2069910 | A | G | 67/141/109 | 0.43 | 0.11 |  | <1 |  |  |  |  |  |
| 127894661 | rs2069912 | G | A | 29/124/165 | 0.29 | 0.41 |  | 0 |  |  |  |  |  |
| 127894742 | rs2069914 | A | G | 28/125/165 | 0.29 | 0.58 |  | <0.03 |  |  |  |  |  |
| 127894885 | rs2069916 | A | G | 35/135/145 | 0.33 | 0.70 |  | <2 |  |  |  |  |  |
| 127895796 | rs2069918 | A | G | 22/109/188 | 0.24 | 0.28 |  | <0.04 |  |  |  |  |  |
| 127896116 | rs2069920 | G | A | 50/145/121 | 0.39 | 0.55 |  | <2 |  |  |  |  |  |
| 127899464 | rs2069924 | A | G | 36/138/142 | 0.33 | 0.80 |  | <2 |  |  |  |  |  |
| 127900384 | rs2069928 | A | C | 13/93/211 | 0.19 | 0.46 |  | <1 |  |  |  |  |  |
| 127902039 | rs2069931 | A | G | 41/135/142 | 0.34 | 0.32 |  | <1 |  |  |  |  |  |
| 127902158 | rs2069933 | A | G | 43/131/145 | 0.34 | 0.13 |  | <0.04 |  |  |  |  |  |
| 127901240 | rs5937 | G | A | 33/135/151 | 0.31 | 0.70 |  | ,<0.04 |  |  |  |  |  |
| 127890289 | rs908787 | G | C | 4/29/286 | 0.058 | 0.015 |  | <0.04 |  |  |  |  |  |
| 127897988 | rs971207 | A | G | 42/134/142 | 0.34 | 0.26 |  | <1 |  |  |  |  |  |

*** Genotype Counts (Minor Homozygotes/ Heterozygotes/ Major Homozygotes)**

**Supplementary Table 2. Characteristics of EPCR tag- SNPs**

| **POSITION** | **SNP** | **Allele 1** | **Allele 2** | **Genotype Counts* (Overall)** | **Minor Allele Frequency** | **Hardy-Wienberg ( Chi-Square) p** | **Missingness (%)** |  |  |  |  |  |  |
| --- | --- | --- | --- | --- | --- | --- | --- | --- | --- | --- | --- | --- | --- |
| 33226150 | rs2069948 | G | A | 66/156/94 | 0.45 | 0.91 | <1.5 |  |  |  |  |  |  |
| 33227425 | rs2069951 | A | G | 0/31/289 | 0.05 | 1 | 0 |  |  |  |  |  |  |
| 33227612 | rs2069952 | G | A | 65/156/96 | 0.45 | 0.91 | <1 |  |  |  |  |  |  |
| 33228215 | rs867186 | G | A | 0/55/264 | 0.08 | 0.14 | <0.04 |  |  |  |  |  |  |
| 33228294 | rs9574 | C | G | 65/149/94 | 0.45 | 0.73 | 3.7% |  |  |  |  |  |  |

*** Genotype Counts (Minor Homozygotes/ Heterozygotes/ Major Homozygotes)**

Supplementary Table 3. Characteristics of Thrombomodulin Gene tag- SNPs

| **Position on Chromosome 20** | **SNP** | **Allele 1** | **Allele 2** | **Genotype Counts* (Overall)** | **Minor Allele Frequency** | **Hardy Wienberg (Chi-Square) P** | **Missingness (%)** |  |  |  |  |  |  |
| --- | --- | --- | --- | --- | --- | --- | --- | --- | --- | --- | --- | --- | --- |
| 22975621 | rs1042580 | G | A | 49/156/114 | 0.40 | 0.81 | <0.04 |  |  |  |  |  |  |
| 22974496 | rs1962 | G | A | 21/105/194 | 0.23 | 0.21 | 0 |  |  |  |  |  |  |
| 22973295 | rs2007363 | A | C | 16/95/209 | 0.20 | 0.22 | 0 |  |  |  |  |  |  |
| 22979523 | rs3176118 | O | X | 3/54/259 | 0.09 | 0.75 | <1 |  |  |  |  |  |  |
| 22975413 | rs3176123 | C | A | 14/87/217 | 0.18 | 0.18 | <0.07 |  |  |  |  |  |  |

*** Genotype Counts (Minor Homozygotes/ Heterozygotes/ Major Homozygotes)**

**ACKNOWLEDGEMENTS**

NATIONAL INSTITUTES OF HEALTH

NATIONAL HEART, LUNG, AND BLOOD INSTITUTE ARDS NETWORK

The following persons and institutions participated in the FACTT trial:

Steering Committee Chair ― G.R. Bernard;

Clinical Coordinating Center ― D.A. Schoenfeld, B.T. Thompson, N. Ringwood, C. Oldmixon, F. Molay, A. Korpak, R. Morse, D. Hayden, M. Ancukiewicz, A. Minihan; Protocol-Review Committee ― J.G.N. Garcia, R. Balk, S. Emerson, M. Shasby, W.Sibbald;

Data Safety and Monitoring Board ― R. Spragg, G. Corbie-Smith, J. Kelley, K. Leeper, A.S. Slutsky, B. Turnbull, C. Vreim;

National Heart, Lung, and Blood Institute ― A.L. Harabin, D. Gail, P. Lew, M. Waclawiw;

ARDS Clinical Trials Network Consultant ― P. Parsons;

Clinical Centers ―

University of Washington, Harborview ― L. Hudson, K. Steinberg, M. Neff, R. Maier, K. Sims, C. Cooper, T. Berry-Bell, G. Carter, L. Andersson;

University of Michigan ― G.B. Toews, R.H. Bartlett, C. Watts, R. Hyzy, D. Arnoldi, R. Dechert, M. Purple;

University of Maryland ― H. Silverman, C. Shanholtz, A. Moore, L. Heinrich, W. Corral;

Johns Hopkins University ― R. Brower, D. Thompson, H. Fessler, S. Murray, A. Sculley; Cleveland Clinic Foundation ― H.P. Wiedemann, A.C. Arroliga, J. Komara, T. Isabella, M. Ferrari;

University Hospitals of Cleveland ― J. Kern, R. Hejal, D. Haney;

MetroHealth Medical Center ― A.F. Connors;

University of Colorado Health Sciences Center ― E. Abraham, R. McIntyre, F. Piedalue; Denver Veterans Affairs Medical Center ― C. Welsh;

Denver Health Medical Center ― I. Douglas, R. Wolkin;

St. Anthony Hospital— T. Bost, B. Sagel, A. Hawkes;

Duke University ― N. MacIntyre, J. Govert, W. Fulkerson, L. Mallatrat, L. Brown, S. Everett, E. VanDyne, N. Knudsen, M. Gentile;

University of North Carolina ― P. Rock, S. Carson, C. Schuler, L. Baker, V. Salo;

Vanderbilt University ― A.P. Wheeler, G. Bernard, T. Rice, B. Christman, S. Bozeman, T. Welch;

University of Pennsylvania ― P. Lanken, J. Christie, B. Fuchs, B Finkel, S. Kaplan, V. Gracias, C.W. Hanson, P. Reilly, M.B. Shapiro, R. Burke, E. O’Connor, D. Wolfe; Jefferson Medical College ― J. Gottlieb, P. Park, D.M. Dillon, A. Girod, J. Furlong; LDS Hospital ― A. Morris, C. Grissom, L. Weaver, J. Orme, T. Clemmer, R. Davis, J. Gleed, S. Pies, T. Graydon, S. Anderson, K. Bennion, P. Skinner;

McKay-Dee Hospital ― C. Lawton, J. d’Hulst, D. Hanselman;

Utah Valley Regional Medical Center ― K. Sundar, T. Hill, K. Ludwig, D. Nielson; University of California, San Francisco ― M.A. Matthay, M. Eisner, B. Daniel, O. Garcia;

San Francisco General ― J. Luce, R. Kallet;

University of California, San Francisco, Fresno ― M. Peterson, J. Lanford;

Baylor College of Medicine ― K. Guntupalli, V. Bandi, C. Pope;

Baystate Medical Center ― J. Steingrub, M. Tidswell, L. Kozikowski; Louisiana State University Health Sciences Center ― B. deBoisblanc, J. Hunt, C. Glynn, P. Lauto, G. Meyaski, C. Romaine;

Louisiana State University Earl K. Long Center ― S. Brierre, C. LeBlanc, K. Reed; Alton-Ochsner Clinic Foundation ― D. Taylor, C. Thompson;

Tulane University Medical Center ― F. Simeone, M. Johnston, M. Wright;

University of Chicago ― G. Schmidt, J. Hall, S. Hemmann, B. Gehlbach, Vinayak, W. Schweickert;

Northwestern University ― J. Dematte D’Amico, H. Donnelly;

University of Texas Health Sciences Center ― A. Anzueto, J. McCarthy, S. Kucera, J. Peters, T. Houlihan, R. Steward, D. Vines;

University of Virginia ― J. Truwit, A.F. Connors, M. Marshall, W. Matsumura, R. Brett; University of Pittsburgh ― M. Donahoe, P. Linden, J. Puyana, L. Lucht, A. Verno;

Wake Forest University ― R.D. Hite, P. Morris, A. Howard, A. Nesser, S. Perez;

Moses Cone Memorial Hospital ― P. Wright, C. Carter-Cole, J. McLean;

St. Paul’s Hospital, Vancouver ― J. Russell, L. Lazowski, K. Foley;

Vancouver General Hospital ― D. Chittock, L. Grandolfo;

Mayo Foundation ― M. Murray.
